# Learning ensembles of process-based models for high accurately evaluating the one-hundred-year carbon sink potential of China’s forest ecosystem

**DOI:** 10.1016/j.heliyon.2023.e17243

**Published:** 2023-06-17

**Authors:** Zhaosheng Wang, Renqaing Li, Qingchun Guo, Zhaojun Wang, Mei Huang, Changjun Cai, Bin Chen

**Affiliations:** a Key Laboratory of Ecosystem Network Observation and Modeling, National Data Center for Ecological Sciences, Institute of Geographic Sciences and Natural Resources Research, CAS, China; b School of Geography and Environment, Liaocheng University, Liaocheng 252000, China; c Key Laboratory of Animal Ecology and Conservation Biology, Institute of Zoology, Chinese Academy of Sciences, Beijing, 100101, China; d Gansu Wuwei Ecological and Environmental Monitoring Center, Wuwei City, Gansu Province, 733000, China

**Keywords:** Learning ensembles of process-based models, Random forest model, Forest ecosystem, Carbon sink potential

## Abstract

China’s forests play a vital role in the global carbon cycle through the absorption of atmospheric CO_2_ to mitigate climate change caused by the increase of anthropogenic CO_2_. It is essential to evaluate the carbon sink potential (CSP) of China’s forest ecosystem. Combining NDVI, field-investigated, and vegetation and soil carbon density data modeled by process-based models, we developed the state-of-the-art learning ensembles model of process-based models **(**the multi-model random forest ensemble (MMRFE) model) to evaluate the carbon stocks of China’s forest ecosystem in historical (1982–2021) and future (2022–2081, without NDVI-driven data) periods. Meanwhile, we proposed a new carbon sink index (CSindex) to scientifically and accurately evaluate carbon sink status and identify carbon sink intensity zones, reducing the probability of random misjudgments as a carbon sink. The new MMRFE models showed good simulation results in simulating forest vegetation and soil carbon density in China (significant positive correlation with the observed values, r = 0.94, P < 0.001). The modeled results show that a cumulative increase of 1.33 Pg C in historical carbon stocks of forest ecosystem is equivalent to 48.62 Bt CO_2_, which is approximately 52.03% of the cumulative increased CO_2_ emissions in China from 1959 to 2018. In the next 60 years, China’s forest ecosystem will absorb annually 1.69 (RCP45 scenario) to 1.85 (RCP85 scenario) Bt CO_2_. Compared with the carbon stock in the historical period, the cumulative absorption of CO_2_ by China’s forest ecosystem in 2032–2036, 2062–2066, and 2077–2081 are approximately 11.25–39.68, 110.66–121.49 and 101.31–111.11 Bt CO_2,_ respectively. In historical and future periods, the medium and strong carbon sink intensity regions identified by the historical CSindex covered 65% of the total forest area, cumulative absorbing approximately 31.60 and 65.83–72.22 Bt CO_2_, respectively. In the future, China’s forest ecosystem has a large CSP with a non-continuous increasing trend. However, the CSP should not be underestimated. Notably, the medium carbon sink intensity region should be the priority for natural carbon sequestration action. This study not only provides an important methodological basis for accurately estimating the future CSP of forest ecosystem but also provides important decision support for future forest ecosystem carbon sequestration action.

## 1 Introduction

As the largest carbon pool in terrestrial ecosystem, forest ecosystem account for approximately 80% of the total carbon stock of terrestrial ecosystem [[1], [2], [3], [4]]. It absorbs atmospheric CO_2_ through plant photosynthesis, which plays an important role in reducing the rise of atmospheric CO_2_ concentrations and mitigating the greenhouse effect [[5], [6], [7]]. In addition, changes in forest ecosystem carbon stock will also affect the climate-change process of global and regional carbon emission reduction [5,6]. Therefore, the changes in the carbon sink potential of forest ecosystem have attracted the attention of natural carbon emission reduction actions.

Currently, the assessment of the carbon sink potential (CSP) of forest ecosystem has become a hot issue in carbon neutrality research [4,[8], [9], [10]]. However, how can the change in forest ecosystem carbon stock be accurately estimated? It is not only a key scientific and technological issue that limits the precision of natural carbon emission reduction actions, but also a difficult issue in the research of land carbon budget assessment. In addition, clarifying the macro pattern, spatial characteristics, and evolution trend of carbon sinks in forest ecosystem will improve the precision in the scientific assessment of terrestrial carbon sink status, and is beneficial to optimize the current implementation program of natural carbon sink enhancements. Noteworthy, as “carbon peak” and “carbon neutrality” have become the strategic goals of China’s future development [11,12], scientifically and accurately assessing CSP of forest ecosystem in China has an extremely important social application value and practical significance.

As an extremely complex ecosystem, the CSP estimation of the forest ecosystem is naturally a complex scientific problem. At present, many relevant studies have laid a cognitive foundation for understanding the CSP evaluation of China’s forest ecosystem. However, due to the differences in estimation methods (such as the biomass method, stock method, biomass inventory method, micrometeorology method, box method, model simulation method, and stable isotope method), there are also large inconsistencies among the estimation results. In addition, the estimation of carbon sinks for forest ecosystem focuses more on the changes in vegetation carbon stock (for example, biomass and stock estimation methods) and less on the changes in forest soil carbon stock, which ignores the carbon fixation role of the forest ecosystem as a whole. This may miscalculate the carbon sink of forest ecosystem. Since both vegetation and soil systems have carbon uptake and emission processes, it may overestimate the carbon sink capacity of forest ecosystem if we only focus on the change in carbon sequestration of vegetation and ignore the change in soil carbon stock.

Currently, there are also great differences in the CSP assessment results of forest ecosystem. For example, how much anthropogenic CO_2_ emissions will be offset by the carbon sink of China’s forest ecosystem in the future? [13]. This still lacks convincing results. In addition, the current mainstream method for determining the carbon source or sink of the ecosystem ignores the continuous stability characteristic of the shift of a carbon source to sink in a given period, which is not enough to scientifically answer the question of whether the ecosystem is a carbon source or sink. For example, in a single year using the simple method of the first-order forward difference to calculate the inter-annual change of carbon stock to judge the status of carbon sinks (that is, if the difference is greater than zero, it is a sink, and vice versa) or simply using the sign change in negative-to-positive of NEP or NBP to judge (that is, if the sign is positive, it is a sink, and vice versa). As these inter-annual changes in the ecosystem, carbon components are also affected by extreme climate events (such as drought, cold waves, etc.), they fluctuate between positive and negative numbers, which undoubtedly increases the uncertainty of the determination of the ecosystem’ carbon source or sink status, resulting in the erroneous judgment of the ecosystem carbon source/sink status. In summary, how to improve the accuracy of carbon stock estimations of forest ecosystem, and scientifically judge their carbon source/sink status are the key issues for scientifically assessments of the CSP of China’s forest ecosystem.

Notably, advanced machine learning (ML) methods not only enhance the accuracy of carbon estimation of terrestrial ecosystems [[14], [15], [16]], but also enrich the technical methods [17,18]. Researchers have explored various ML models such as random forests [[19], [20], [21]], artificial neural networks [22], and support vector machines [23,24] to estimate carbon components. Additionally, studies have investigated the use of remote sensing data, including LiDAR and satellite imagery, in combination with ML algorithms for carbon mapping [19,21]. ML models can be adapted to integrate new data sources or variables, leading to improved accuracy and reliability of carbon stock estimates over time by identifying complex relationships between environmental variables. Furthermore, ML models are being developed for real-time monitoring of carbon fluxes, allowing for timely identification of both carbon sequestration opportunities and potential threats to ecosystem health. Naturally, as an important ML method, learning ensemble method also has excellent performance in estimating carbon stock of terrestrial ecosystems [14,15,21].

To accurately estimate carbon stock, and scientifically judge their carbon source/sink status of the CSP of China’s forest ecosystem, as a method of learning ensembles of process-based models for effectively improving the estimation accuracy of terrestrial ecosystem carbon stock [20,21], we employ a multi-model random forest ensemble (MMRFE) model (see detailed information in Section 2.4) for evaluating CSP of China’s forest ecosystem in the historical (1982–2021) and future (2022–2081) periods. Meanwhile, we also proposed a new carbon sink index method that takes into account the characteristics of the continuous stability of carbon sink function of ecosystem for scientifically identifying the carbon sink status of the forest ecosystem. Further, we use the index to divide the carbon sink intensity zones. Finally, we evaluate the historical and future CSPs of China’s forest ecosystem in different carbon sink intensity zones.

## 2 Data and methods

### 2.1 Forest carbon density data from process-based models

To construct and drive a new MMRFE model, we used forest vegetation carbon (labeled as cVeg) and soil carbon density (labeled as cSoil) datasets simulated by models from CMIP6 and CMIP5. For the historical period (1982–2021), we use seven-model data of CMIP6 (BCC-CCSM2-MR (labeled as M_1_), CESM2-WACCM (M_2_), CMCC-CM2-SR5 (M_3_), CMCC-ESM2 (M_4_), EC-Earth3-CC (M_5_), EC-Earth3-Veg (M_6_), and TaiESM1 (M_7_), downloaded from https://esgf-node.llnl.gov/search/cmip6/). Since the CMIP6 SSP2-4.5 scenario is closest to the real world, therefore these model outputs of SSP2-4.5 forcings were used in the later simulations for the 2016–2021 period. For the future period (2022–2081), we also employed seven-model data of RCP45 and RCP85 scenarios from CMIP5 (HadGEM2-CC ((labeled as CM_1_), HadGEM2-ES (CM_2_), IPSL-CM5A-LR (CM_3_), IPSL-CM5A-MR (CM_4_), IPSL-CM5B-LR (CM_5_), MPI-ESM-LR (CM_6_), and MPI-ESM-MR (CM_7_), downloaded from https://esgf-node.llnl.gov/search/cmip5/). The CMIP5 outputs in the period of 2010–2081 were used. Table 1 also lists a short introduction of these process-based model.

**Table 1:** Short introduction for process-based models.

Model name	Land model	Spatial resolution	Country	Data source
BCC-CSM2-MR	BCC-AVIM2	1° × 1°	China	CMIP6
CESM2-WACCM	CLM4.0	1° × 1°	America	CMIP6
CMCC-CM2-SR5	CLM4.5	1° × 1°	Italy	CMIP6
CMCC-ESM2	CLM4.5	1° × 1°	Italy	CMIP6
EC-Earth3-CC	LPJ-GUESS	1° × 1°	European Union	CMIP6
EC-Earth3-Veg	LPJ-GUESS	1° × 1°	European Union	CMIP6
TaiESM1	CLM4	1° × 1°	China	CMIP6
HadGEM2-CC	TRIFFID	1.875° × 1.25°	UK	CMIP5
HadGEM2-ES	TRIFFID	1.875° × 1.25°	UK	CMIP5
IPSL-CM5A-LR	ORCHIDEE	3.75° × 1.88°	France	CMIP5
IPSL-CM5A-MR	ORCHIDEE	2.50° × 1.26°	France	CMIP5
IPSL-CM5B-LR	ORCHIDEE	3.75° × 1.88°	France	CMIP5
MPI-ESM-LR	JSBACH	1.88° × 1.88°	Germany	CMIP5
MPI-ESM-MR	JSBACH	1.88° × 1.88°	Germany	CMIP5

Using the CDO (Climate Data Operator, version 1.9.9, from https://code.mpimet.mpg.de/projects/cdo) software, we extract spatial ModcVeg and ModcSoil values of China’s forest from these datasets and then use a bilinear interpolation method to downscale them to the grid surface with a spatial resolution of 0.01*0.01°. Using only the 4 nearest pixel values in diagonal directions from a given pixel, the bilinear interpolation can fast and simply calculated the appropriate value of that pixel [25]. This method may reduce some of the visual distortion caused by resizing an image, and is broadly applied in digital image processing.

### 2.2 Forest observation carbon density data from field-investigated plots

To train and test the new model, we use 5-year (2011–2015) field-investigated plot vegetation carbon (labeled as ObscVeg) and soil carbon (labeled as ObscSoil) density datasets from Tang et al. [26]. These data have been used to estimate the carbon pool of China’s terrestrial ecosystem [20,21,[27], [28], [29]]. In this study, using a bilinear interpolation technology, the ObscVeg and ObscSoil values of 7800 forest plots were interpolated to a grid surface with a spatial resolution of 0.01 * 0.01° in 2010, respectively.

### 2.3 A new method for reproducing a set of 1000-m NDVI data during 1982–2021

Since the normalized difference vegetation index (NDVI) is obtained by remote sensing observation, it can truly reflect the spatial distribution patterns of vegetation. In addition, it has a potential relationship with vegetation biomass and soil organic carbon, so NDVI was used as a covariate to control the spatial modal change of vegetation carbon and soil carbon. To correct the spatial distribution of forest carbon density, we proposed a new method to reproduce a set of long-term continuous 1000-m NDVI annual time series. Hence, we combined 8 km GIMMS3g NDVI (1982–2015) and 1 km MODIS NDVI (2000–2021) datasets to reproduce 1000-m annual NDVI data from 1982 to 2021. The GIMMS3g NDVI with a temporal resolution of two weeks was downloaded from https://iridl.ldeo.columbia.edu/SOURCES/.NASA/.ARC/.ECOCAST/.GIMMS/.NDVI3g/.v1p0/. The biweekly MODIS NDVI datasets were from https://lpdaac.usgs.gov/products/mod13a2v006/. First, the monthly NDVI dataset was reconstructed using the maximum value synthesis (MVC) method [30]. Then, using a bilinear interpolation method, the GIMMS3g NDVI was interpolated onto the grid surface of 0.01*0.01° with the same spatial resolution as MODIS NDVI. The annual NDVI value is a 12-month average NDVI value in the current year. Notably, a prior information about the distribution of vegetation provided by the resampled GIMMS3g NDVI values to help reduce the probability of non-vegetation being treated as plants in the constructed 1000-m NDVI datasets. The detailed method of reproducing 1000-m NDVI during 1982–2021 is showed following equation (1):

(1) $$\begin{array}{l} {\text{NDVI}}_{y,ix,jx}=\frac{1}{12}\sum_{m=1}^{m=12}NDVI_{m,ix,jx}\\ NDVI_{m,ix,jx}>0.1\\ ANDVI_{G,ix,jx}=\frac{1}{22}\sum_{y=1}^{y=12}NDVI_{G,ix,jx}\\ ANDVI_{M,ix,jx}=\frac{1}{22}\sum_{y=1}^{y=12}NDVI_{M,ix,jx}\\ V_{ix,jx}=\frac{ANDVI_{G,ix,jx}}{ANDVI_{M,ix,jx}}\\ NDVI_{ix,jx}=\left(1+\frac{NDVI_{G,i,j}-ANDVI_{M,ix,jx}}{{\text{ANDVI}}_{M,ix,jx}}\right)\times \frac{NDVI_{G,i,j}}{V_{ix,jx}},i<ix,j<jx\\ i=h+ix\cdot \Delta_{i},h<\Delta_{i}\\ j=v+jx\cdot \Delta_{j},v<\Delta_{j} \end{array}$$

where i (or ix) and j (or jx) refer to the $i_{th}$ (or ${ix}_{th}$) row and $j_{th}$ (or ${jx}_{th}$) column pixel in the image data, respectively. $\Delta_{i}$ is $\frac{ix}{i}$; $\Delta_{j}$ is $\frac{jx}{j}\text{.}$ y and m are the year and month, respectively. $NDVI_{G}$ and $NDVI_{M}$ refer to GIMM3g and MODIS NDVI, and ANDVI is the annual NDVI value.

We have demonstrated that the 1000-m NDVI is available (Supplementary Fig. 1). Supplementary Fig. 1 shows that the annual variation trend of 1000-m NDVI is consistent with that of GIMMS3g and MODIS NDVI. And 1000-m NDVI is significantly correlated with GIMMS3g and MODIS NDVI. These important information proofs that in this study we constructed 1000-m NDVI has a high accuracy and precision.

### 2.4 Model-building methods for evaluating carbon stock

As a type of ensemble techniques, random forests (RF) can integrate a large number of randomly constructed decision trees into a forest, and use the average method to merge the predicted values of all the trees to get a more accurate prediction value [31,32]. RF’s input variables are searched to find the best split for each node, and its algorithm has an excellent ability to process large data with accuracy [33]. For example, Wang et al. [20] reported that a mulitmodel random forests ensemble model (MMRFE) can effectively improve the estimation accuracy of the terrestrial vegetation carbon density. Furtherly, Wang [21] also confirmed that the MMRFE model driven by a combing remote sensed NDVI with outputs from process-based models can perfectly estimate the carbon density of forest litter at the national scale. These reports proof that the MMRFE model can effectively improve terrestrial carbon ensemble evaluation. Due a large discrepancy in evaluating carbon stocks from process-based models, for accurately measuring China’s forest carbon stocks, we propose a theoretical model approach using RF learning ensemble to estimate carbon stocks from 1982 to 2081. The approach is showed following equations (2), (3), (4):

(2) $$\begin{array}{l} M_{cVeg,MMRFE,hist}=f\left(\overset{X_{2010}}{\overset{⌢}{NDVI,M_{1,cVeg},M_{2,cVeg},M_{3,cVeg},M_{4,cVeg},M_{5,cVeg},M_{6,cVeg},M_{7,cVeg}}},\overset{Y_{2010}}{\overset{⌢}{ObscVeg}}\right)\\ M_{cSoil,MMRFE,hist}=f\left(\overset{X_{2010}}{\overset{⌢}{NDVI,M_{1,cSoil},M_{2,cSoil},M_{3,cSoil},M_{4,cSoil},M_{5,cSoil},M_{6,cSoil},M_{7,cSoil}}},\overset{Y_{2010}}{\overset{⌢}{ObscSoil}}\right)\\ cVeg_{i,MMRFE,hist}=M_{cVeg,MMRFE,hist}\left(\overset{X_{i,i\in \left[1982,2021\right]}}{\overset{⌢}{NDVI,M_{1,cVeg},M_{2,cVeg},M_{3,cVeg},M_{4,cVeg},M_{5,cVeg},M_{6,cVeg},M_{7,cVeg}}}\right)\\ cSoil_{i,MMRFE,hist}=M_{cSoil,MMRFE,hist}\left(\overset{X_{i,i\in \left[1982,2021\right]}}{\overset{⌢}{NDVI,M_{1,cSoil},M_{2,cSoil},M_{3,cSoil},M_{4,cSoil},M_{5,cSoil},M_{6,cSoil},M_{7,cSoil}}}\right) \end{array}$$

(3) $$\begin{array}{l} M_{cVeg,MMRFE,future}=f\left(\overset{X_{2010}}{\overset{⌢}{CM_{1,cVeg},CM_{2,cVeg},CM_{3,cVeg},CM_{4,cVeg},CM_{5,cVeg},CM_{6,cVeg},CM_{7,cVeg}}},\overset{Y_{2010}}{\overset{⌢}{ObscVeg}}\right)\\ M_{cSoil,MMRFE,future}=f\left(\overset{X_{2010}}{\overset{⌢}{CM_{1,cSoil},CM_{2,cSoil},CM_{3,cSoil},CM_{4,cSoil},CM_{5,cSoil},CM_{6,cSoil},CM_{7,cSoil}}},\overset{Y_{2010}}{\overset{⌢}{ObscSoil}}\right)\\ cVeg_{i,MMRFE,future}=M_{cVeg,MMRFE,future}\left(\overset{X_{i,i\in \left[2011,2081\right]}}{\overset{⌢}{CM_{1,cVeg},CM_{2,cVeg},CM_{3,cVeg},CM_{4,cVeg},CM_{5,cVeg},CM_{6,cVeg},CM_{7,cVeg}}}\right)\\ cSoil_{i,MMRFE,future}=M_{cSoil,MMRFE,future}\left(\overset{X_{i,i\in \left[2011,2081\right]}}{\overset{⌢}{CM_{1,cSoil},CM_{2,cSoil},CM_{3,cSoil},CM_{4,cSoil},CM_{5,cSoil},CM_{6,cSoil},CM_{7,cSoil}}}\right) \end{array}$$

(4) $$C_{total}=cVeg+cSoil$$

where M_cVeg_ and M_cSoil_ are from CMIP6 outputs, CM_cVeg_ and CM_cSoil_ are from CMIP5 outputs, M_cVeg, MMRFE,hist_ and M_cVeg, MMRFE,future_ refer to refer to corresponding building models for historical and future periods respectively, C_total_ refers to total carbon density.

First, based on the coordinate information of field-investigated plots, at the corresponding coordinate points, we extracted the NDVI and output values of process-based models in 2010 were extracted. Here, according the (2), (3), based on based on scikit-learn (version 0.23, from https://scikit-learn.org/stable/), employing a training strategy of bootstrap samples (75%) and a hyperparameter optimization method, we use NDVI, 2010 ObscVeg (or ObscSoil) and CMIP6 model-based cVeg (or cSoil) outputs to build the new learning ensemble models of MMRFE, and then run the optimal MMRFE models to re-estimate China’s forest cVeg and cSoil during 1982–2021. Also, due no future NDVI, only using 2010 ObscVeg (or ObscSoil) and CMIP5 model-based cVeg (or cSoil) outputs, we build and train the optimal MMRFE models for reevaluating the total carbon stocks in future (RCP45 and RCP85 scenarios) periods (2022–2081).

### 2.5 Statistical validation

Comparing model results and observation values, we use the Taylor chart statistical method [34] to test the simulation performance of the new MMRFE models. The correlation coefficient (R), root mean square difference (RMSD), and standard deviation (SD) from the method give a concise statistical result to illustrate the matching of patterns between model-based and filed-investigated values. The performance of the MMRFE models was evaluated by these variables. R is often used to quantify the mode similarity between the model (m) and the observed value (o) to detect whether M and o are linearly related. Its value is calculated following equation (5):

(5) $$R=\frac{\frac{1}{n}\sum_{n=1}^{N}\left(M_{n}-\bar{M_{n}}\right)\left(O_{n}-\bar{O_{n}}\right)}{\sigma_{M}\sigma_{O}}$$

where σ is the standard deviation, and the calculation method is showed following equation (6):

(6) $$\sigma_{X}=\frac{1}{N}\sqrt{\sum_{n=1}^{N}{\left(X-\bar{X}\right)}^{2}}\left(X=M\;or\;O\right)$$

RMSD is the error distance between the model and the observation data, which is defined following equation (7):

(7) $$RMSD={\left(\frac{1}{N}\sum_{n=1}^{N}{\left[\left(M_{n}-\bar{M_{n}}\right)\left(O_{n}-\bar{O_{n}}\right)\right]}^{2}\right)}^{0.5}$$

### 2.6 Identifying methods for carbon sink and its intensity zone

To reduce the probability of erroneous judgment on the carbon sink status of forest ecosystem, we propose the forest ecosystem carbon sink index (CSindex) based on the forest ecosystem carbon stock data in historical periods, taking into account the continuous stability characteristic of the shift of a carbon source-to-sink in a given period. According to the historical carbon sink index data, using the K-clustering method in machine learning methods, the Chinese forest ecosystem are divided into three categories: weak sink, medium sink, and strong carbon sink areas. The carbon sink index is calculated following equation (8):

(8) $$\left\{ \begin{array}{l} \Delta C_{i,j,k}=C_{i,j,k+1}-C_{i,j,k}\\ n_{i,j,k}=1,\Delta C_{i,j,k}>0\\ C\;\mathrm{Sin}\;dex_{i,j}=\frac{1}{N}\sum_{k=0}^{N}n_{i,j,k}\cdot 100\%，C\;\mathrm{Sin}\;dex_{i,j}\in \left(0,1\right) \end{array}\right.$$

In the above formula, k is the kth year, n is the 39th year, $C_{i,j,k}$ is the carbon stock at the ith and jth grid points in the kth year, $\Delta C_{i,j,k}$ is the carbon increment, and CSindex is the carbon sink index. The 39 is not given arbitrarily, but is determined by the length of the remote sensing observation period.

## 3 Results

### 3.1 Performances of new MMRFE models for estimating China’s forest carbon density

**Fig. 1:**
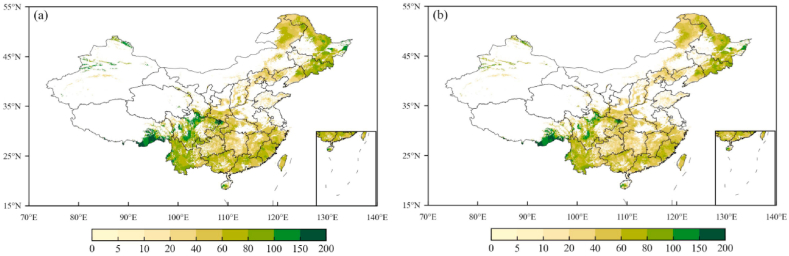
Comparison of observed (a) and simulated (b) vegetation carbon density in forest ecosystem in 2010. (a) shows the spatial interpolation results of the observed values. (b) shows the simulation results of the new MMRFE model. Unit: Mg C/ha.

**Fig. 2:**
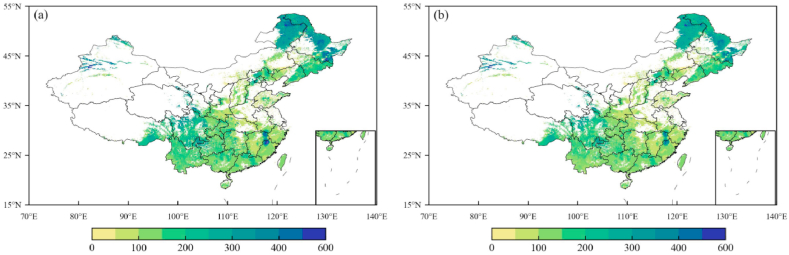
Comparison of observed (a) and simulated (b) soil carbon density in forest ecosystem in 2010. (a) shows the spatial interpolation results of the observed values. (b) shows the simulation results of the new MMRFE model. Unit: Mg C/ha.

We employed the method in Section 2.3 to develop the new MMRFE models for estimating China’s forest carbon density, and run the trained models to re-estimate forest ModcVeg and ModcSoil value over China in 2010. Compared to the spatial difference between these observed and modeled values (Fig. 1, Fig. 2), we found that the new MMRFE models have a good simulation performance in re-estimating forest carbon density. For forest vegetation carbon density, the statistical results in Fig. 1 show that the national average ModcVeg value of 42.9 Mg C/ha simulated by the new model (Fig. 1b) is −0.66% slightly lower than the observed value (43.1 Mg C/ha, Fig. 1a), and the median value of ModcVeg (39.8 Mg C/ha) is just −4.55% lower than that of ObsVeg (41.7 Mg C/ha).

Meanwhile, the compared results of soil carbon density in Fig. 2 show that the national average ModcSoil value of 147.0 Mg C/ha simulated by the new model (Fig. 2b) is −0.12% slightly lower than the ObscSoil value (Fig. 2a), and the median value of ModcSoil (39.8 Mg C/ha) is just 1.4% higher than that of ObsSoil (128.2 Mg C/ha).

**Fig. 3:**
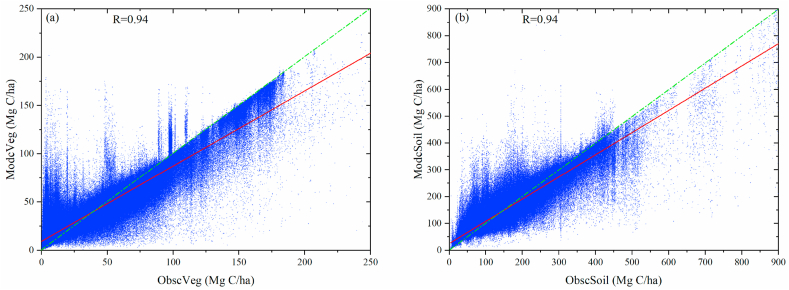
(a) The correlation coefficient between grid-to-grid ModcVeg and ObscVeg of forest ecosystem over China in 2010. (b) is the same as (a) but for soil carbon density of forest ecosystem.

Additionally, we further investigated the correlation coefficients between grid-to-grid modeled and observed carbon density values (Fig. 3). The results show that both ModcVeg (Fig. 3a) and ModcSoil (Fig. 3b) values significantly correlated ObscVeg and ObscSoil values at the 99% confidence level (both Rs are 0.94). The results imply that the vegetation carbon and soil carbon density simulated by the new MMRFE model are highly consistent with the field-investigated values.

**Fig. 4:**
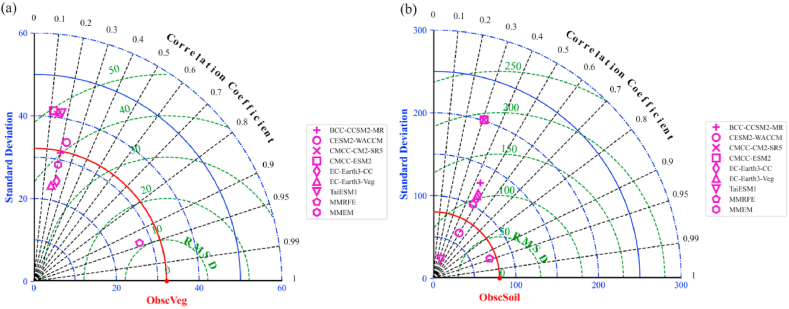
Taylor diagram for terrestrial vegetation (a, ModcVeg versus ObscVeg) and soil (b, ModcSoil versus ObscSoil) carbon density validation in China. MMEM stands for a multi-model ensemble average model.

Furtherly, using a Taylor chart analysis method to detect the relationships among MMRFE-based, raw-model-based, and filed-investigated carbon density values, we test the simulation performances of the new MMRFE model on simulating forest vegetation carbon (Fig. 4a) and soil carbon (Fig. 4b) density. Fig. 4a shows that ModcVeg and ObscVeg have the highest correlation coefficient (r = 0.94) and the smallest root mean square error (RMSE = 11.4 Mg C/ha); Fig. 4b also shows that ModcSoil and ObscSoil have the highest correlation coefficient (r = 0.94) and the smallest root mean square error (RMSE = 26.6 Mg C/ha). Meanwhile, the more detailed test information from Table 2 also shows that the MMRFE models gain a high model skill score.

**Table 2:** The statistical comparison of model results across China in 2010.

Classes	Feature Variables	Obs	M_1_	M_2_	M_3_	M_4_	M_5_	M_6_	M_7_	M_8_	M_9_
cVeg	SD	32.12	31.66	34.48	40.70	41.51	24.82	23.55	41.28	28.76	27.18
	RMSD	0.00	41.66	45.44	53.21	54.19	36.39	36.32	63.76	41.87	11.40
	R	1.00	0.20	0.23	0.14	0.12	0.22	0.18	0.17	0.20	0.94
cSoil	SD	80.30	128.57	63.10	201.16	201.43	114.22	112.63	24.98	101.68	72.32
	RMSD	0.00	117.64	85.39	297.54	298.18	117.48	115.98	92.31	114.83	26.65
	R	1.00	0.44	0.50	0.31	0.31	0.48	0.47	0.35	0.47	0.94

*Note:* ‘Obs’ is field-investigated value. M_1_–M_9_ are BCC-CCSM2-MR, CESM2-WACCM, CMCC-CM2-SR5, CMCC-ESM2, EC-Earth3-CC, EC-Earth3-Veg, TaiESM1, MMEM and MMRFE model, respectively. MMEM stands for a multi-model ensemble average model. The SD and RMSE units are Mg C/ha.

For example, refereeing to field-investigated values, compared with 3-feature (R, RMSD, and SD) among raw models and MMEM (a multi-model ensemble average model) model, both MMRFE-modeled ModcVeg and ModcSoil values have the highest R, the smallest RMSD, and smaller SD value. The above information suggests that the simulation accuracy of the MMRFE-modeled forest carbon density is the best, compared with that of the BCC-CCSM2-MR, CESM2-WACCM, CMCC-CM2-SR5, CMCC-ESM2, EC-Earth3-CC, EC-Earth3-Veg, TaiESM1, and MMEM models.

**Fig. 5:**
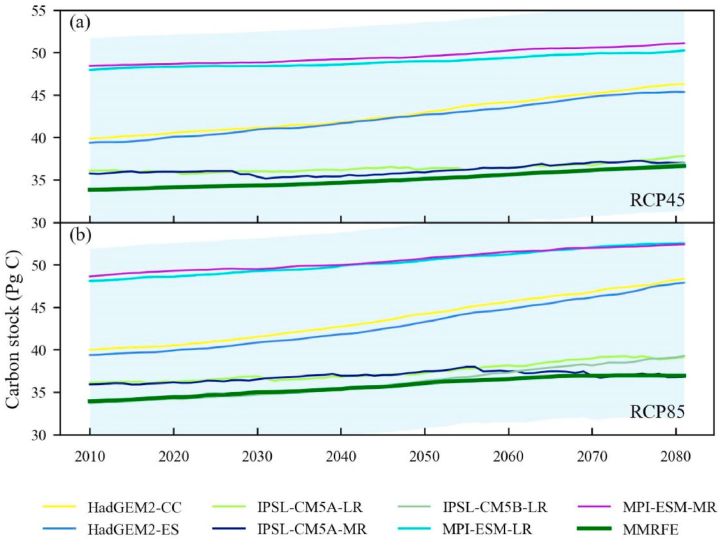
The annual changes of total carbon stocks from CMIP5 raw models and MMRFE model under RCP45 (a) and RCP85 (b) scenarios. The blue shading represents 95% confidence distribution intervals, which are detected by a mean ± 1.96 standard deviation from raw models' outputs. (For interpretation of the references to colour in this figure legend, the reader is referred to the Web version of this article.)

Meanwhile, we compared the annual changes of total carbon stocks from MMRFE and CMIP5 raw models under RCP45 (Fig. 5a) and RCP85 (Fig. 5b) during 2010–2081. Fig. 5 shows that the annual variation trends in total carbon stock simulated by MMRFE model are consistent with the increase trends of CMIP5 model, and the MMRFE values lie within the 95% confidence interval. Table 3 also show that the slopes of all models' outputs are greater than 7.0 Pg C/y, which suggests that all models' results have an increase trend. The total carbon stock values of MMRFE significantly correlate with that of CMIP5 models (their R values are greater than 0.8 under p < 0.001 level). Obviously, the data of China’s forest carbon stock in the future period modeled by MMRFE model have a strong availability.

**Table 3:** The statistical comparison of model results across China during 2010–2081.

Scenarios	Elements of comparison	CM_1_	CM_2_	CM_3_	CM_4_	CM_5_	CM_6_	CM_7_	CM_8_
RCP45	Slope (Pg C/y)	10.44	10.81	39.44	26.78	22.32	31.63	25.02	24.18
	R	0.999	0.995	0.911	0.843	0.996	0.995	0.997	1.000
RCP85	Slope (Pg C/y)	7.94	7.84	19.02	27.78	12.13	14.92	17.83	20.03
	R	0.986	0.978	0.950	0.808	0.979	0.990	0.989	1.000

*Note:* CM_1_–CM_8_ are HadGEM2-CC, HadGEM2-ES, IPSL-CM5A-LR, IPSL-CM5A-MR, IPSL-CM5B-LR, MPI-ESM-LR, MPI-ESM-MR and MMRFE model. Slope is the linear change slope of total carbon storage from 2010 to 2081. R is a correlation coefficient between MMRFE and CMIP5 raw models' outputs.

To sum up, this information implies that in this study the developed MMRFE models can be used to accurately estimate China’s forest vegetation and soil carbon density, and are available learning ensemble method of process-based models for evaluating the carbon sink potential of forest ecosystem.

### 3.2 Spatial and temporal changes in historical forest CSP

**Fig. 6:**
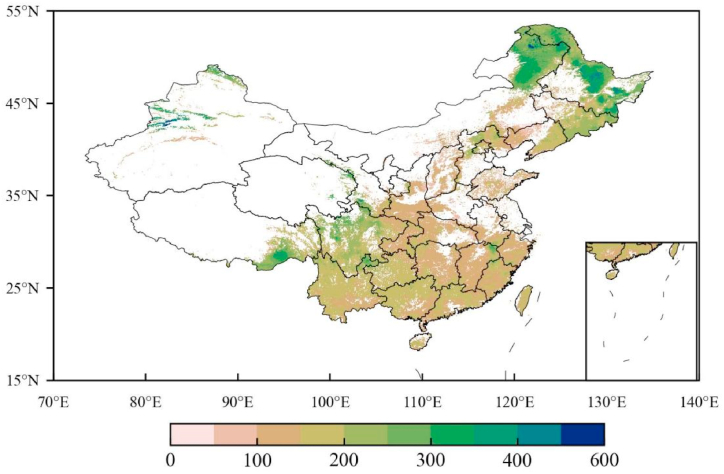
The spatial distribution of multiyear average ecosystem carbon density (ECD) of forest in China during 1982–2021. Forest ECD is the sum of vegetation carbon and soil carbon density. Unit: Mg C/ha.

We use the trained MMRFE models with a high skill score to re-estimate the forest vegetation and soil carbon density in China from 1982 to 2021, respectively. Then, a direct sum value of the two carbon densities is defined as an ecosystem carbon density (ECD) of the forests. Furtherly, we investigate the spatial distribution of the 40-year average ECD (Fig. 6). The statistical results in Fig. 6 show that the national average ECD is 175.1 Mg C/ha and the covered area with an ECD higher than the value is approximately 41.9% of the total forest coverage, but for the area with an ECD lower than the value is 58.1%.

**Fig. 7:**
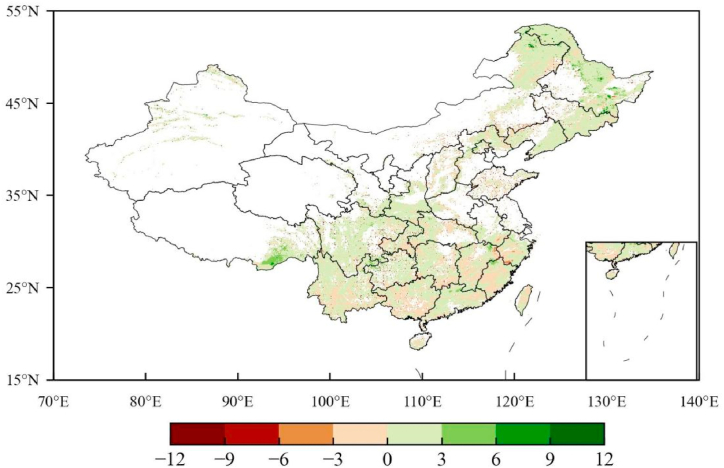
The change rate of forest ECD in China from 1982 to 2021. Unit: Mg C ha^−1^ yr^−1^ Use a slope of linear regression to detect the change rate of ECD.

We also use a slope of linear regression to detect the spatial distribution of the ECD change rate in the past 40 years (Fig. 7). Over 40 years the national average slope is 0.07 Mg C ha^−1^ yr^−1^. The covered area with an increasing trend (that is slope >0 Mg C ha^−1^ yr^−1^) is approximately 51.1% of the total area.

Meanwhile, the regions with a slope great equal to 0.07 Mg C ha^−1^ yr^−1^ is approximately 51.0% of the total forest area, but where with a lower value are 49%. Additionally, the 40-year average forest ecosystem carbon stock is 33.3 ± 0.54 Pg C/yr. Over 40 years, a significant increase trend (r = 0.71, p < 0.01) in forest ecosystem carbon stock also suggests that China’s forests have a large CSP. Compared with 32.92 Pg C forest carbon stock in 1982, it increased by 3.6% (1.17 Pg C) in 2021 in 2021. Table 4 further analyzes the changes in 5-span carbon stock. Compared with the average carbon stock from 1982 to 1986, China’s forest carbon stock has increased by 1.33 Pg C in the past 40 years, with an average annual increase of 0.033 Pg C/yr. This suggests that China’s forest ecosystem has absorbed approximately 1.21 Bt CO_2_ annually, which accounts for approximately 77.56% of China’s annual average increase of 1.56 Bt CO_2_ during 1959–2018. The 40-year cumulative amount of CO_2_ absorbed by the forest ecosystem is 48.62 Bt, which accounts for approximately 52.03% of China’s accumulated emissions of 93.45 Bt CO_2_ from 1959 to 2018. The above information directly shows that the historical CSP of China’s forest is large.

**Table 4:** The potential carbon sink potential (CSP) from forest ecosystem in China during the historical period (1982–2021).

Period	Carbon stock (Pg C/a)	SD (Pg C/a)	Carbon Increment (Pg C)	Cumulative Carbon Increment (Pg C)	Cumulative Absorbed CO2 (BT CO_2_)
1982–1986	32.89	0.04	0.00	0.00	0.00
1987–1991	32.84	0.07	−0.07	−0.05	−1.92
1992–1996	32.87	0.10	0.05	−0.02	−0.83
1997–2001	32.95	0.05	0.08	0.06	2.18
2002–2006	33.16	0.10	0.21	0.27	9.85
2007–2011	33.76	0.46	0.60	0.62	22.74
2012–2016	33.67	0.50	−0.09	0.78	28.58
2017–2021	34.21	0.17	0.55	1.33	48.62

### 3.3 Historical spatial pattern of CSP and its change

**Fig. 8:**
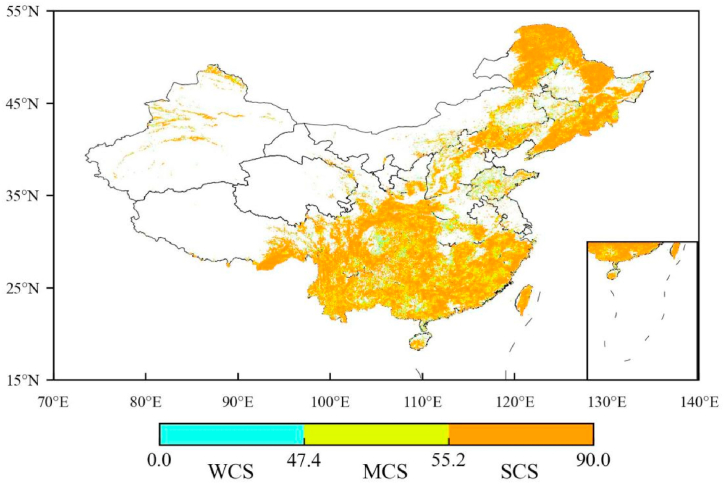
Carbon sink index partitioning of forest ecosystem in China from 1982 to 2021. The legend represents the CSindex values. The WCS, MCS and SCS are the weak, medium and strong carbon sink region, respectively.

We also investigate the historical carbon sink status of the forest ecosystem (Fig. 8), using the CSindex method (more detailed information refer to Section 2.6). The results in Fig. 8 show that a medium carbon sink (MCS) with the range 47.38%≤CSindex<55.23% dominates the current spatial pattern of forest carbon sink, which covered 45% of the total forest area. However, a strong (with the range of CSindex≥55.23%) and weak carbon sink (with the range of 0<CSindex<47.38%) distribution only covered 20% and 35% of the total forest area, respectively. The regions of strong carbon sink (SCS) are where the number of an increase in carbon stock in 40 years is more than 23 years but the weak carbon sink (WCS) regions with a number less than 19 years. The number of the MCS regions are in a range of 19–23 years.

Based on the historical CSP data in Table 4, we further evaluate the carbon stocks and the amount of cumulatively absorbed CO_2_ from the atmosphere in different carbon sink regions. In the past 40 years, the average carbon stocks of forest ecosystem in the WCS, MCS, and SCS regions were 11.66, 14.98, and 6.66 Pg C, respectively. And their cumulatively increased carbon stocks were 0.47, 0.60, and 0.26 Pg C, respectively. Meanwhile, over 40 years, their corresponding amounts of cumulatively absorbed CO_2_ were 17.02, 21.88, and 9.72 Bt, which can offset 18.2%, 23.41%, and 10.4% of the total cumulatively increased CO_2_ emission over China during 1959–2018, respectively. The above information implies that current China’s forest ecosystem is a medium carbon sink region with a large CSP.

### 3.4 Temporal changes in future forest CSP under RCP45 and RCP85 scenarios

**Fig. 9:**
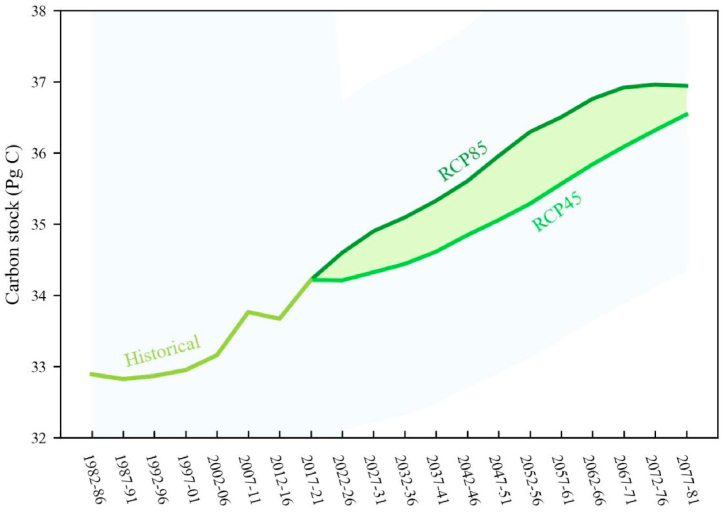
The 5-pan change in carbon stock of China’s forest ecosystem in historical and future (RCP45 and RCP85 scenarios) periods. The shaded green part depicts the amplitude of variations. The shaded blue part depicts an error range given by the simulated variables from all the models. (For interpretation of the references to colour in this figure legend, the reader is referred to the Web version of this article.)

We continue to employ the newly trained MMRFE model to estimate the future forest CSP under RCP45 and RCP85 scenarios (Fig. 9). Fig. 9 shows that during one hundred years significant increase trends (r > 0.98, p < 0.001) in forest carbon stocks appear. Under RCP45 and RCP85 scenarios, carbon stocks increased from 34.31 to 36.54 and 36.94 Pg C, respectively. In the next 60 years, Supplementary Fig. 2a shows that the total cumulatively increased carbon stocks of forest ecosystem are 3.23 (RCP45) and 3.63 (RCP85) Pg C. In the contemporaneous future, Supplementary Fig. 2b also shows that the total cumulatively absorbed CO_2_ by forest ecosystem are 118.34 (RCP45) and 133.06 (RCP85) Bt. Supplementary Fig. 2 also depicts the amplitude of these variations (see the shaded part in the figure). Assuming the value of RCP85 as the upper limit of these variables, their maximum amplitudes occur during 2052–2056. During 2027–2076, carbon stocks have a larger average amplitude of 0.81 Pg C. Notably, during 2027–2066 the average value of the ratio of the amplitude to the RCP45 CO_2_ absorption is greater than 50%. The important tip suggests that the period of 2027–2066 may be a period of a high probability that China’s forest ecosystem has a large CSP.

We investigate the CSP in different carbon sink zones in the future period (Table 5, Table 6, Table 7). Table 5, Table 6, Table 7 show that under the RCP45 and RCP85 scenarios, compared with the average value of forest ecosystem from 1982 to 2021, the carbon stocks from 2032 to 2036 will increase by 1.126 and 1.779 Pg C, respectively, which is equivalent to the cumulative absorption of 41.294 and 65.225 Bt CO_2_ by forest ecosystem. From 2062 to 2066, the carbon stocks of the forest ecosystem will increase by 2.525 and 3.447 Pg C, respectively, which is equivalent to the cumulative absorption of 92.588 and 126.398 Bt CO_2_. In the next 60 years (2077–2081), forest carbon stock will increase by 3.227 and 3.629 Pg C, respectively, which is equivalent to 118.338 and 133.064 Bt CO_2_ absorbed by the forest ecosystem. This means that in the next 60 years, China’s forest ecosystem will absorb approximately 1.97–2.22 Bt CO_2_ annually. Especially in the MCS region forest ecosystem will absorb 53.251–59.878 Bt CO_2_, and in the SCS region forest ecosystem will cumulatively absorb 23.669–26.614 Bt CO_2_. The above information suggests that in the future period China’s forest ecosystem also has a large CSP.

**Table 5:** Carbon stocks of different carbon sink zones in China’s forest ecosystem in the future period (2022–2081) (Pg C).

Period	China		WCS Zone		MCS Zone		SCS Zone	
	RCP45	RCP85	RCP45	RCP85	RCP45	RCP85	RCP45	RCP85
2022_2026	34.21	34.60	11.97	12.11	15.39	15.57	6.84	6.92
2027_2031	34.32	34.90	12.01	12.21	15.44	15.70	6.86	6.98
2032_2036	34.44	35.09	12.05	12.28	15.50	15.79	6.89	7.02
2037_2041	34.84	35.60	12.19	12.46	15.68	16.02	6.97	7.12
2042_2046	35.05	35.96	12.27	12.58	15.77	16.18	7.01	7.19
2047_2051	35.05	35.96	12.27	12.58	15.77	16.18	7.01	7.19
2052_2056	35.28	36.29	12.35	12.70	15.88	16.33	7.06	7.26
2057_2061	35.56	36.50	12.45	12.78	16.00	16.43	7.11	7.30
2062_2066	35.84	36.76	12.54	12.87	16.13	16.54	7.17	7.35
2067_2071	36.08	36.92	12.63	12.92	16.24	16.61	7.22	7.38
2072_2076	36.32	36.96	12.71	12.94	16.34	16.63	7.26	7.39
2077_2081	36.54	36.94	12.79	12.93	16.44	16.62	7.31	7.39

*Note:* According to Fig. 8, China’s forest ecosystem can be divided into weak, moderate, and strong carbon sink zone.

**Table 6:** Cumulatively increased carbon stocks in different carbon sink zones in the future period (2022–2081) in China in the future (2022–2081) (Pg C).

Period	China		WCS Zone		MCS Zone		SCS Zone	
	RCP45	RCP85	RCP45	RCP85	RCP45	RCP85	RCP45	RCP85
2022_2026	0.896	1.284	0.313	0.449	0.403	0.578	0.179	0.257
2027_2031	1.009	1.587	0.353	0.555	0.454	0.714	0.202	0.317
2032_2036	1.126	1.779	0.394	0.623	0.507	0.800	0.225	0.356
2037_2041	1.531	2.289	0.536	0.801	0.689	1.030	0.306	0.458
2042_2046	1.742	2.644	0.610	0.925	0.784	1.190	0.348	0.529
2047_2051	1.742	2.644	0.610	0.925	0.784	1.190	0.348	0.529
2052_2056	1.972	2.981	0.690	1.043	0.887	1.341	0.394	0.596
2057_2061	2.251	3.190	0.788	1.116	1.013	1.435	0.450	0.638
2062_2066	2.525	3.447	0.884	1.206	1.136	1.551	0.505	0.689
2067_2071	2.772	3.607	0.970	1.262	1.248	1.623	0.554	0.721
2072_2076	3.005	3.646	1.052	1.276	1.352	1.641	0.601	0.729
2077_2081	3.227	3.629	1.129	1.270	1.452	1.633	0.645	0.726

*Note:* The statistical results were based on the historical period (1982–2021) of China’s forest ecosystem carbon stock.

**Table 7:** Cumulatively absorbed CO_2_ by China’s forest ecosystem from the atmosphere in different carbon sink zones in the future period (2022–2081) (Bt CO_2_).

Period	Total absorbed CO_2_		WCS Zone		MCS Zone		SCS Zone	
	RCP45	RCP85	RCP45	RCP85	RCP45	RCP85	RCP45	RCP85
2022_2026	32.845	47.066	11.496	16.473	14.779	21.178	6.571	9.415
2027_2031	37.004	58.199	12.951	20.369	16.650	26.188	7.402	11.641
2032_2036	41.294	65.225	14.453	22.829	18.581	29.350	8.260	13.046
2037_2041	56.131	83.934	19.646	29.377	25.258	37.769	11.228	16.788
2042_2046	63.883	96.937	22.359	33.928	28.746	43.620	12.778	19.389
2047_2051	63.883	96.937	22.359	33.928	28.746	43.620	12.778	19.389
2052_2056	72.295	109.310	25.303	38.258	32.531	49.188	14.460	21.863
2057_2061	82.541	116.966	28.889	40.938	37.142	52.634	16.510	23.395
2062_2066	92.588	126.398	32.406	44.239	41.663	56.878	18.519	25.281
2067_2071	101.660	132.272	35.581	46.295	45.746	59.521	20.334	26.456
2072_2076	110.175	133.695	38.561	46.793	49.577	60.162	22.036	26.740
2077_2081	118.338	133.064	41.418	46.572	53.251	59.878	23.669	26.614

*Note:* The results in this Table are converted by multiplying the results in Table 4 by 3.667 × 10.

## 4 Discussion

In this section, we discuss the developed learning ensemble model and its results. Furtherly, based on our results, we also put forward the future directions for improvements in the accurate estimation of carbon sinks.

### 4.1 Advances in simulation of China’s forest carbon density by MMRFE model

In this study, we use remote sensing observation data to drive the developed learning ensemble models to re-estimate the carbon stocks of the Chinese forest ecosystem. The method not only optimizes the spatial distribution mode of traditional process-model simulation results but also improves the accuracy and precision of the dynamic estimation of carbon stocks of the forest ecosystem. Because most process models driven by the static vegetation coverage data usually ignore the succession between forest ecosystem types over a long-term scale. Hence, these results from the models cannot correctly depict the distribution status of forest ecosystem types in historical periods, which further increases the estimation uncertainty of forest ecosystem carbon stock. In addition, it is noteworthy that the proposed carbon sink index method takes into account the continuous stability characteristic of the shift of a carbon source-to-sink in a given period, which is enough to more objectively identify the carbon sink spatial pattern of China’s forest ecosystem. The new index helps to reduce the probability of erroneous judgment of the ecosystem’s carbon source/sink status.

**Fig. 10:**
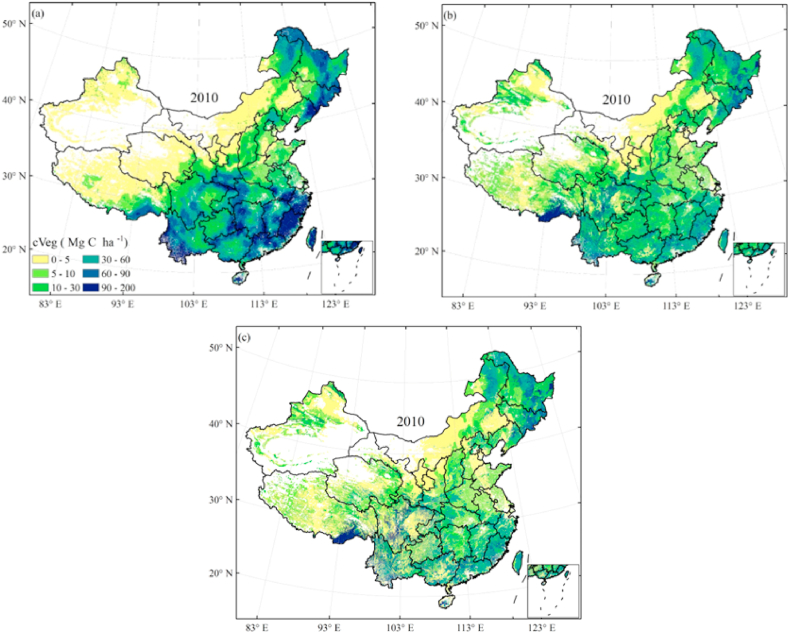
Comparison of cVeg from MsTMIP (a), MMRFE (b) model and observation (c) in 2010. (a) is an arithmetic mean result of multiple models. MsTMIP models include BIOME-BGC, CLASSCTEM-N, CLM4, CLM4VIC, DLEM, ISAM, TEMP6 and TRIPLEX-GHG model. Using the bilinear interpolation method, MsTMIP cVeg result were downscaled to a 0.01-deg spatial resolution by using CDO (Climate Data Operator, Version 1.9.9, from https://code.mpimet.mpg.de/projects/cdo) software.

Compared with most of the existing studies focusing on the carbon sequestration of forest vegetation, this study considers the overall CSP of forest ecosystem and more accurately evaluates the CSP of forest ecosystem. Furtherly, to test a robust availability of MMRFE model we have compared vegetation carbon density (cVeg) between from MMRFE and the MsTMIP (available download website https://daac.ornl.gov/cgi-bin/dsviewer.pl?ds_id=1225) over China in 2010 (Fig. 10). Fig. 10ashows that the spatial distribution of cVeg from MsTMIP is obviously different from that of the observation (Fig. 10c). But the spatial modes (Fig. 10b) from MMRFE are highly similar to the observed values. The result suggests that MMRFE model has a strong prediction in terrestrial carbon density.

Compared to other research findings, we also test the availability of MMRFE cVeg and cSoil (Table 8). Table 8 shows that both MMRFE cVeg (42.04 ± 15.09 Mg C/ha) and cSoil (101.00 ± 39.46 Mg C/ha) value lie within the 95% confidence interval. The result also shows that MMRFE cVeg and cSoil are available. The above all indicate that MMRFE has an advance in simulating forest vegetation carbon and soil in China.

**Table 8:** Comparative verification of vegetation (cVeg) and soil (cSoil) carbon density of forest.

cVeg (Mg C/ha)	Sources	cSoil (Mg C/ha)	Sources
55.69	[26]	88.7	[35]
37.8	[36]	98.5	[37]
38.7	[36]	88.7	[29]
39.18	[38]	79.5	[26]
34.63	This study	119.2	This study
46.27	[39]	131.4	[40]

### 4.2 Shortcomings and prospects

However, the estimated CSP of this study is slightly lower than that of others in the future [41,42]. Most of the results are estimated by using explosion models (for example, logistic equation or other power exponential model methods) with a monotonically increasing property. Consequently, these simulation results inevitably have an increasing trend. The simulation results of such models are reliable in the short term, but in the long term, this intrinsic increase tendency will undoubtedly lead to an overestimation of CSP.

Using the MMRFE model to simulate the long-term dynamic process of variables, whether the model is convergent, or whether the high value is underestimated or the low value is overestimated, still needs further research. Since based on only limited fixed-point observation data and coarser vegetation type data (it is difficult to accurately identify a mixed coniferous-broadleaf forest), it is impossible to estimate the carbon stock changes in forest ecosystem at the national scale. And then it is impossible to accurately estimate the carbon stock change characteristics of a mixed coniferous-broadleaf forest. At the same time, it is difficult to obtain high-precision NDVI values matched with observation stations, which may miscalculate the NDVI of mixed forests, thus increasing the uncertainty of the assessment results of CSP of forest ecosystem.

## 5 Conclusion

It is essential to accurately evaluate the carbon sink potential (CSP) from China’s forest ecosystem. Combing NDVI, field-investigated and vegetation and soil carbon density data modeled by process-based models, we developed the state-of-the-art of learning ensembles model of process-based models to evaluate China’s forest ecosystem carbon stocks in historical (1982–2021) and future (2022–2081, without NDVI-driven data) periods. Meanwhile, we proposed a new carbon sink index (CSindex) to scientifically and accurately evaluate carbon sink status and identify carbon sink intensity zones, reducing the probability of random misjudgments as a carbon sink. The model comparison results show that the new MMRFE models perform good simulation results in simulating forest vegetation and soil carbon density in China (significant positive correlation with the observed values, r = 0.94, p < 0.001). Since the new learning ensemble models have the highest simulation skill scores, the developed MMRFE models can effectively improve the estimation accuracy of vegetation carbon and soil carbon density of forest ecosystem. Over 1982–2021, the annual change results of carbon stocks in forest ecosystem show that there is a significant increase trend (r = 0.71, p < 0.01) in carbon stock. Meanwhile, the cumulatively increased carbon stock is 1.33 Pg C, equivalent to 48.62 Bt of the cumulative absorption of atmospheric CO_2_, which is approximately 52.03% of China’s total cumulatively increased CO_2_ emissions from 1959 to 2018. In the next 60 years, China’s forest ecosystem will absorb annually 169 (RCP45 scenario) to 185 (RCP85 scenario) Mt CO_2_. Compared with the carbon stock in the historical period, the cumulative absorption of CO_2_ by China’s forest ecosystem in 2032–2036, 2062–2066, and 2077–2081 are approximately 11.25–39.68, 110.66–121.49 and 101.31–111.11 Bt CO_2,_ respectively. This suggests that China’s forest ecosystem has a large CSP in the future. In historical and future periods, the MCS and SCS regions identified by the historical CSindex covered 65% of the total forest area, cumulative absorbing approximately 31.60 and 65.83–72.22 Bt CO2, respectively. Shortly, in the future period, China’s forest ecosystem has a large CSP with a non-continuous increasing trend, which makes an important contribution to the continuous removal of atmospheric CO_2_. Hence, the CSP should not be underestimated. Notably, the MCS region should be the priority for natural carbon sequestration action.

In short, this study not only provides an important methodological basis for accurately estimating the future CSP of forest ecosystem but also provides important decision support for future forest ecosystem carbon sequestration action.

## Author contribution statement

Zhaosheng Wang and Mei Huang conceived and designed the study. Zhaosheng Wang, Ren Qiang Li, Qingchun Guo, Zhaojun Wang, and Changjun Cai performed the study. Zhaosheng Wang, Ren Qiang Li, Qingchun Guo, Zhaojun Wang, and Changjun Cai analyzed and interpreted the data. Zhaosheng Wang, Mei Huang, Renqiang Li, Changjun Cai and Bin Chen contributed data and analysis tools. Zhaosheng Wang, Ren Qiang Li, Qingchun Guo, Zhaojun Wang and Bin Chen wrote the paper.

## Data availability statement

Data will be made available on request. Competing Interest.

## Declaration of competing interest

The authors declare that they have no known competing financial interests or personal relationships that could have appeared to influence the work reported in this paper
