# Supplementary material for: Learning ensembles of process-based models for high accurately evaluating the one-hundred-year carbon sink potential of China’s forest ecosystem
Source: Heliyon. 2023 Jun 17;9(6):e17243. doi: 10.1016/j.heliyon.2023.e17243 (PMC10333463; doi:10.1016/j.heliyon.2023.e17243)
Supplement: Multimedia component 1 [file mmc1.docx]

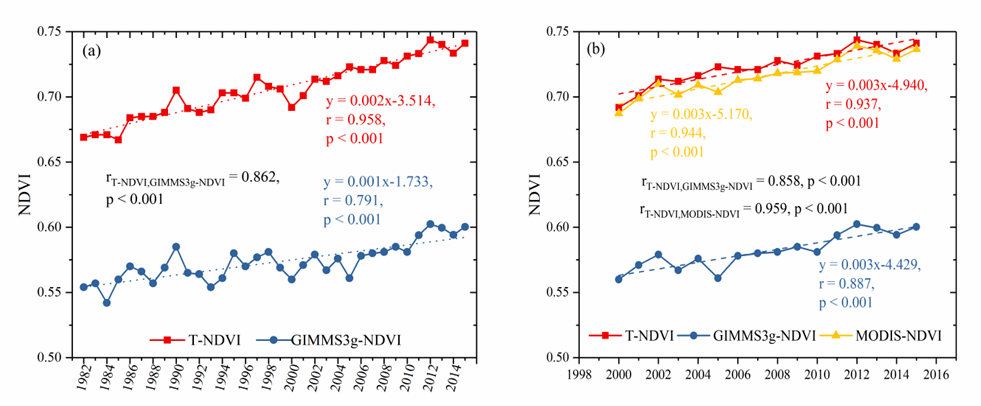


**Supplementary Figure 1.** Comparisons of China’s forest NDVI between T-NDVI and GIMMS3g, MODIS NDVI. T-NDVI constructed by using Section 2.3 method were used in this study.


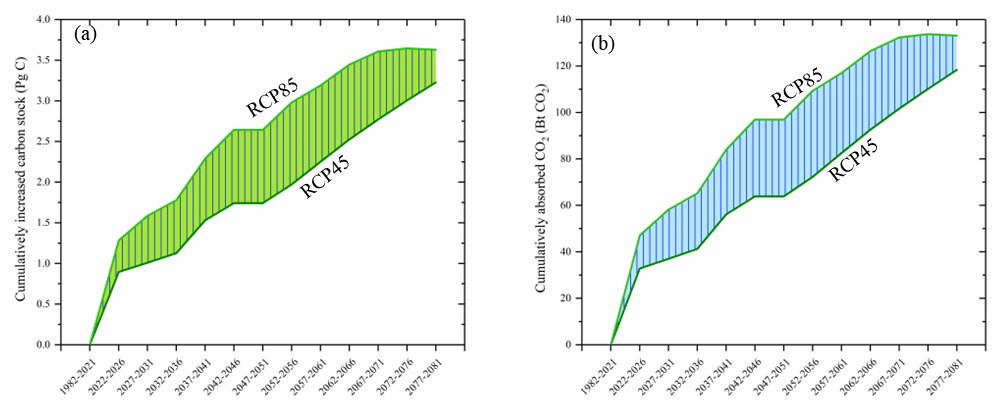


**Supplementary Figure 2.** The 5-pan change in cumulatively increased carbon stock (a) and cumulatively absorbed CO_2_ (b) of China’s forest ecosystem in future (RCP45 and RCP85 scenarios) periods. The shaded part depicts the amplitude of variations.
